# Claudin-1 and Claudin-3 as Molecular Regulators of Myelination in Leukoaraiosis Patients

**DOI:** 10.6061/clinics/2021/e2167

**Published:** 2021-05-06

**Authors:** Yan Chen, Zheng Zheng, Ainong Mei, Huan Huang, Fan Lin

**Affiliations:** I Shengli Clinical Medical College, Fujian Medical University, Fuzhou, 350001, P.R. China; II Department of Geriatric Medicine, Fujian Provincial Hospital, Fuzhou, 350001, P.R. China; III Fujian Key Laboratory of Geriatrics, Fuzhou, 350001, P.R. China; IV Fujian Provincial center for Geriatrics, Fuzhou, 350001, P.R. China; V Department of Neurology, Fujian Provincial Hospital, Fuzhou, 350001, P.R. China

**Keywords:** Leukoaraiosis, Claudin-1, Claudin-3, Myelination, Oligodendrocyte, Apoptosis

## Abstract

**OBJECTIVES:**

Leukoaraiosis is described as white matter lesions that are associated with cognitive dysfunction, neurodegenerative disorders, etc. Myelin depletion is a salient pathological feature of, and the loss of oligodendrocytes is one of the most robust alterations evident in, white matter degeneration. Recent studies have revealed that claudin proteins are aberrantly expressed in leukoaraiosis and regulate oligodendrocyte activity. However, the roles of claudin-1 and claudin-3 in oligodendrocytes and leukoaraiosis are still not well-defined.

**METHODS:**

Quantitative polymerase chain reaction was used to measure the expression of claudin-1 (*CLDN1*), claudin-3 (*CLDN3*), and myelinogenesis-related genes such as myelin basic protein (*MBP*), proteolipid protein (*PLP*), oligodendrocyte transcription factor 2 (*OLIG2*), and SRY-box transcription factor 10 (*SOX10*) in leukoaraiosis patients (n=122) and healthy controls (n=122). The expression of claudin-1 and claudin-3 was either ectopically silenced or augmented in Oli-neu oligodendrocytes, and colony formation, apoptosis, and migration assays were performed. Finally, the expression of myelin proteins was evaluated by western blotting.

**RESULTS:**

Our results revealed that in addition to SOX10, the expression levels of claudin-1, claudin-3, and myelinogenesis-related proteins were prominently downregulated in leukoaraiosis patients, compared to those in healthy controls. Furthermore, the growth and migration of Oli-neu cells were downregulated upon silencing claudin-1 or claudin-3. However, the overexpression of claudin-1 or claudin-3 resulted in the reduction of the degree of apoptosis in Oli-neu cells. In addition, claudin-1 and claudin-3 promoted the expression of MBP, OLIG2, PLP, and SOX10 at the translational level.

**CONCLUSION:**

Our data has demonstrated that the abnormal expression of claudin-1 and claudin-3 regulates the pathological progression of leukoaraiosis by governing the viability and myelination of oligodendrocytes. These findings provide novel insights into the regulatory mechanisms underlying the roles of claudin-1 and claudin-3 in leukoaraiosis.

## INTRODUCTION

Leukoaraiosis (LA), also defined as white matter lesions, is a neuroimaging manifestation of cerebrovascular disease; it mainly manifests as a diffuse white matter abnormality near the lateral ventricle. LA has become a conventional prognosis in describing cognitive dysfunction, stroke injury, cerebral small vessel disease, and neurodegenerative disorders (1,2). Increasing evidences show that more than 40% of apparently healthy adults over 50 years old present with the characteristics of high-intensity LA (3). LA has gradually become an important global health problem. However, the underlying mechanism of LA is still unclear. Therefore, it is imperative to explore its pathogenesis and seek effective and feasible strategies for its prevention.

The degeneration of white matter causes myelin loss, axonal abnormalities, arteriolosclerosis, and parenchymal changes. Neuropathological research has shown that these changes result from chronic hypoperfusion injury. Myelination disorder is a typical lesion of hypoxic-ischemic white matter damage. Ischemia induces the degeneration and apoptosis of glial cells, which result in the demyelination of nerve fibers and axon damage, causing neuronal damage, cognitive dysfunction, and even dementia (4). Oligodendrocytes are the main myelinating cells in the central nervous system. The plasma membrane of oligodendrocytes wraps around the axon to form a protective and dense myelin sheath, assisting the efficient transmission of bioelectrical signals, and maintaining and protecting the normal function of neurons (5). Oligodendrocyte loss is one of the most robust alterations in LA.

The regulatory mechanisms underlying the action of myelin sheaths are essential for the maintenance and repair of white matter (6). Myelin basic protein (MBP), proteolipid protein (PLP), oligodendrocyte transcription factor 2 (OLIG2), and SRY-box transcription factor 10 (SOX10) are the common regulators of myelinogenesis. MBP and PLP are structural myelin specific proteins that are expressed throughout the myelin sheaths to stabilize the myelin structure. Their expression levels are amplified during myelination and subsequently silenced. These proteins can also be re-expressed after demyelinating episodes, where they contribute to re-myelination (7). OLIG2 and SOX10 are transcription factors that regulate the differentiation and myelination of oligodendrocyte precursor cells (OPCs). OLIG2 deletion-induced hypomyelination specifically contributes to a significant loss of excitatory synapses and functional deficits after chronic hypoxia (8). Myelin sheaths are crucial for the survival and maintenance of the axons and the rapid propagation of the action potential. In oligodendrocytes of the central nervous system, SOX10 is essential for myelination and its maintenance via targeting myelin gene-myelin oligodendrocyte glycoprotein (*MOG*) (9). The myelin sheath is composed of several layers of cytoplasmic membrane from oligodendrocytes. One oligodendrocyte may myelinate over 40 axons (7). Thus, targeting oligodendrocytes affecting myelination and re-myelination may serve as a promising treatment strategy for functional recovery post white matter injury.

Neurological dysfunction in LA patients reflects the damage of the white matter communications channels (10,11). Claudins (CLDNs) are 20-24-kDa tetraspan membrane proteins that function as the structural and functional components of tight junctions and contribute to obliterate the intercellular space (12). Increasing evidences have shown that the members of the claudin family are abnormally expressed during the development of LA. Hypoxic-ischemic encephalopathy induces the loss of tight-junction proteins such as CLDN-1 and Zonula occludens-1 (ZO-1). Human Urinary Kallidinogenase (HUK) protein and mild hypothermia in hypoxic-ischemic encephalopathy are neuroprotective at least in part by rescuing the loss of tight-junctions (13). Pathological conditions (ischemia and hypoxia) downregulate CLDN-1 and CLDN-3; this damages the tightened paracellular barrier and interconnected cells and contributes to tight junction and blood-brain barrier damage (14). According to the earlier studies, the blood brain barrier is crucial in brain homeostasis modulation and exhibits an important role in the development of LA (15,16). Interestingly, it has been shown that CLDN-11 regulates the expression of MBP and PLP, which ensures the accurate formation of compact myelin and supports the axonal integrity of mature oligodendrocytes (17). However, whether CLDN-1 and CLDN-3 could modulate myelin production still remains to be elucidated.

In this study, we have reported the expression levels of CLDN-1 and CLDN-3 in LA patients and compared them to those in healthy individuals. Our data shows that the expression of CLDN-1 and CLDN-3 is vital for oligodendrocyte proliferation, apoptosis, and migration, and the proper maintenance of myelin proteins. These results demonstrate that CLDN-1 and CLDN-3 regulate the expression of MBP, PLP, OLIG2, and SOX10 in oligodendrocytes and thus, affect the progression of LA.

## MATERIALS AND METHODS

### Clinical samples

A total of 122 patients of Chinese ethnicity with LA aged 60-86 years participated in the current study. Of these patients, 64 and 58 were male and female patients respectively. The clinical characteristics with LA based on the MRI (SIEMENS Aera 1.5T) scan have been determined. LA was diagnosed as hyperintensities around the lateral ventricle or subcortical area (T2WI and T2 FLAIR). In addition, age-matched 122 normal elderly without cognitive impairment were screened as normal controls. Biochemical parameters were estimated using an Olympus AU5400 automated analyzer (Olympus Optical Co. Ltd., Tokyo, Japan). All the clinical characteristics of patients and control subjects, such as the age and neurological and brain MRI findings, were collected (Table 1). This study was approved by the Ethics Committee of Fujian Provincial Hospital, People's Republic of China. Written informed consents were obtained from the patients that participated in the study.

### RNA isolation and gene expression

Venous blood was extracted from patients before treatment and stored at −80°C until analysis. The NucleoZOL^®^ (gene, Co., Ltd, Shanghai, China) was utilized for total RNA extraction from blood cells based on the manufacturer’s instructions. The extracted RNA was reverse transcribed by the Reverse Transcription System Kit (Takara, Dalian, China). The synthesized cDNA was amplified by quantitative polymerase chain reaction (PCR) using the HEAL FORCE (Xianggang, China). The reaction conditions were 42°C for 60 min, followed by cooling to 4°C. The resultant cDNA (RT product) was used as a template for subsequent PCR. The master mix prepared for each single real-time (PCR) reaction contained 10 µL qPCR SYBR^®^ Green Master Mix Universal, 10 µL primer (10 µM), 3 µL RNase free water, and 2 µL of each RT product. Forty cycles of PCR amplification were performed, with initial incubation at 95°C for 10 min and final extension at 72°C for 5 min. Each cycle comprised of denaturation at 95°C for 10s, annealing at 60°C for 30s, and extension at 72°C for 30s. The mRNA expression levels of *CLDN-1*, *CLDN-3*, *PLP*, *MBP*, *OLIG2*, and *SOX10* were normalized to those of *GAPDH*. Relative expression of genes was calculated by the formula ΔΔCt = (Ct. _Target_-Ct. _internal reference_) _Patient_ - (Ct. _Target_-Ct. _internal reference_) _Control_, and estimated the fold change in expression as 2^-ΔΔCt^. The RNA primers used for RT-PCR amplification are shown in Table 2.

### Cell culture and transfection

The Oli-neu cells were cultivated in Dulbecco’s modified essential medium (Thermo Fisher Scientific, Waltham, MA, USA) supplemented with 10% fetal bovine serum (Thermo Fisher Scientific), 5% horse serum (Gibco, USA), and N2 supplement (Invitrogen, USA) in a 5% CO_2_ atmosphere at 37°C. The Oli-neu cells were cultivated 24 hours and then transfected by the Lipofectamine 2000 reagent (Invitrogen, USA). For knockdown experiment, the shRNAs targeting CLDN1, CLDN3, or sh-NC (Negative control) were purchased and transfected in the Oli-neu cells. For overexpression assay, the cells were transfected with the CLDN1 or CLDN3 overexpression constructs (pcDNA3.1-CLDN1 or pcDNA3.1-CLDN3). The cells were harvested for the subsequent analysis 48 h post transfection.

### Colony formation assay

The Oli-neu cells were cultured in a 6-well plate. After 48h post transfection, the medium was discarded; then, the cells were gently washed three times with PBS and immediately stained with crystal violet (Sigma-Aldrich, MO, USA) for additional 2-3 h at room temperature. The size of each colony (>50 μm) was detected using the Quantity One^®^ Software (Bio-Rad, Richmond, USA).

### Cell apoptosis detection

The degree of apoptosis of Oli-neu cells was assessed by performing Annexin V fluorescein isothiocyanate (FITC) staining assay. When they attained 80-90% confluence, the transfected cells were harvested and washed three times with PBS. The binding buffer supplemented with an appropriate amount of propidium iodide and Annexin V- FITC was added to the transfected cells (10^6^ cells). Subsequently, the treated cells were cultured for an additional 1h in the dark. Flow cytometry (Beckman Coulter, Brea, USA) was performed to analyze the degree of apoptosis.

### Wound healing assay

Oli-neu cells (5×10^5^) were cultivated in a 6 well plate for subsequent transfection. After 24h, a 200-μL pipette tip was used to scratch the monolayer of the cells and serum-free DMEM medium was added into the wells for 48h. The wound width was photographed with the help of a microscope at 0 and 48h; subsequently, the migration ability of the cells was analyzed by Image J sofware.

### Western blotting

The pre-cold RIPA lysis buffer (Solarbio, Beijing, China) with PMSF was added into the treated cells for 20 min on ice. Subsequently, the suspension was centrifuged at 12000 rpm for 10 min at 4°C. The concentration of proteins in the supernatants (cell lysates) was estimated by using the bicinchoninic acid (BCA) protein assay kit (Epizyme, People's Republic of China). The 10 µL of extracted protein was loaded per well and resolved using the sodium dodecyl sulphate-polyacrylamide gel electrophoresis (SDS-PAGE). The resolved proteins were immediately transferred onto a polyvinylidene difluoride (PVDF) membrane (Millipore, MA, USA). The treated membrane was blocked in blocking buffer (5% skimmed milk in 20 mM Tris-HCl, 150 mM NaCl, 0.1% Tween-20) for 1 hour, washed three times in Tris buffered saline with 0.1% Tween-20 (TBS-T), and incubated with the primary antibodies at 4°C overnight. The following day, they were incubated with the secondary-HRP-antibodies (Protein-Tech, USA) (1:5000) for additional 1 hour at room temperature. Enhanced chemiluminescence (GE Healthcare, USA) assay was used to detect the signal. The primary antibodies used in the study are as follows: anti-CLDN-1 (#4933, 1:1000), anti-CLDN-3 (#83609, 1:1000), anti-MBP (#78896, 1:1000), anti-PLP (#85971, 1:1000), anti-SOX10 (#69661, 1:1000), and anti-actin (#4967, 1:1000) antibodies, which were purchased from Cell Signaling Technology; anti-OLIG2 (sc-293163, 1:500) antibodies were obtained from Santa Cruz Biotechnology.

### Statistical analysis

All the data was analyzed using GraphPad Prism 6.0 and SPSS 20.0. If the data obeyed a normal distribution, the student's t test was used for comparison between the two groups, and one-way ANOVA test was used for the comparison between multiple groups. If data did not obey normal distribution, Mann-Whitney U test was used. *p*<0.05 was considered to be statistically significant. Data are presented as the mean±SD of three independent experiments.

## RESULTS

### Clinical characteristics

**Figure 1:**
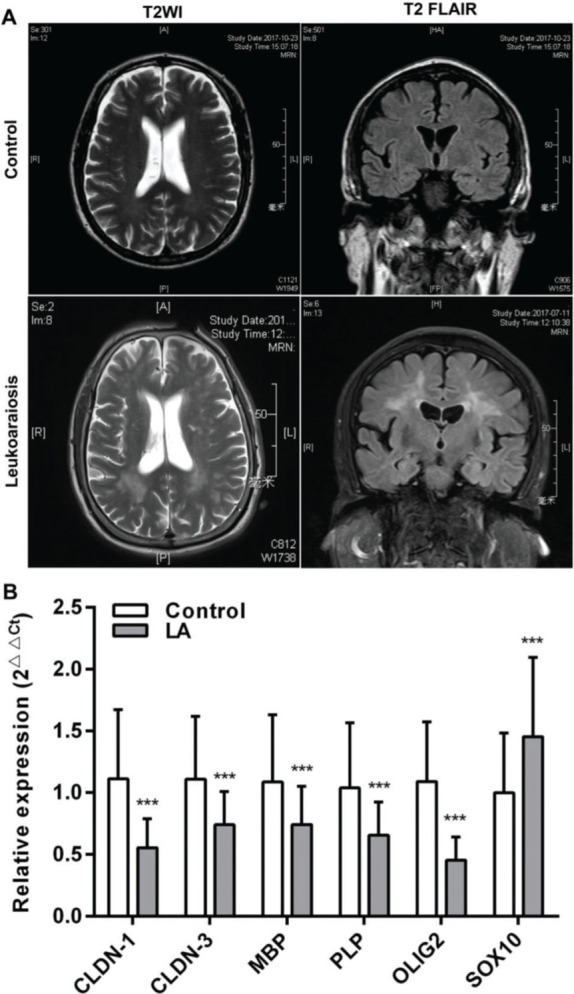
White matter (WM) lesions visualized in normal controls and patients with LA and the relative expression of claudins. (A) The magnetic resonance imaging (MRI) exhibits symmetrical deep lesions located in the periventricular white matter, which showed high signals in T2WI and T2 FLAIR. (B) The expression of claudins (*CLDN-1* and *CLDN-3*) and myelinogenesis-related genes (*MBP*, *PLP*, *OLIG2*, and *SOX10*) in patients with LA. ***p<0.001 *versus* the respective control.

All the patients were clinically diagnosed with LA according to the clinical manifestations presented. The relative biochemical parameters of the LA patients are shown in Table 1. The LA group consisted of 122 patients (52.45% men), with an average age of 72 (66-77) years. The normal control (NC) group included 122 healthy people (61.47%), with an average age of 71 (66.75-76) years. There was no significant difference in age, sex, and the levels of CRP (C-reactive protein), HDL-C (high-density lipoprotein cholesterol), GLU (Fasting blood glucose), and HbA1c (Glycosylated hemoglobin) between the two groups (*p*>0.05). Compared with the NC group, the levels of TC (total cholesterol), LDL-C (low-density lipoprotein cholesterol), TG (triglyceride), UA (uric acid), and Hcy (total plasma homocysteine), and the scores of MMSE (the mini-mental state examination), MoCA (montreal cognitive assessment scale), and Fazeka in LA patients showed a statistically significant difference *(p<*0.05). In these patients, symmetrical abnormalities in deep white matter (WM) were observed in MRI images (Figure 1A). The qRT-PCR revealed that the mRNA levels of CLDN-1, CLDN-3, MBP, PLP, and OLIG2 were remarkably downregulated and those of SOX10 were remarkably upregulated (*p*<0.001) (Figure 1B).

### CLDN-1 and CLDN-3 play an important functional role in Oli-neu cells

**Figure 2:**
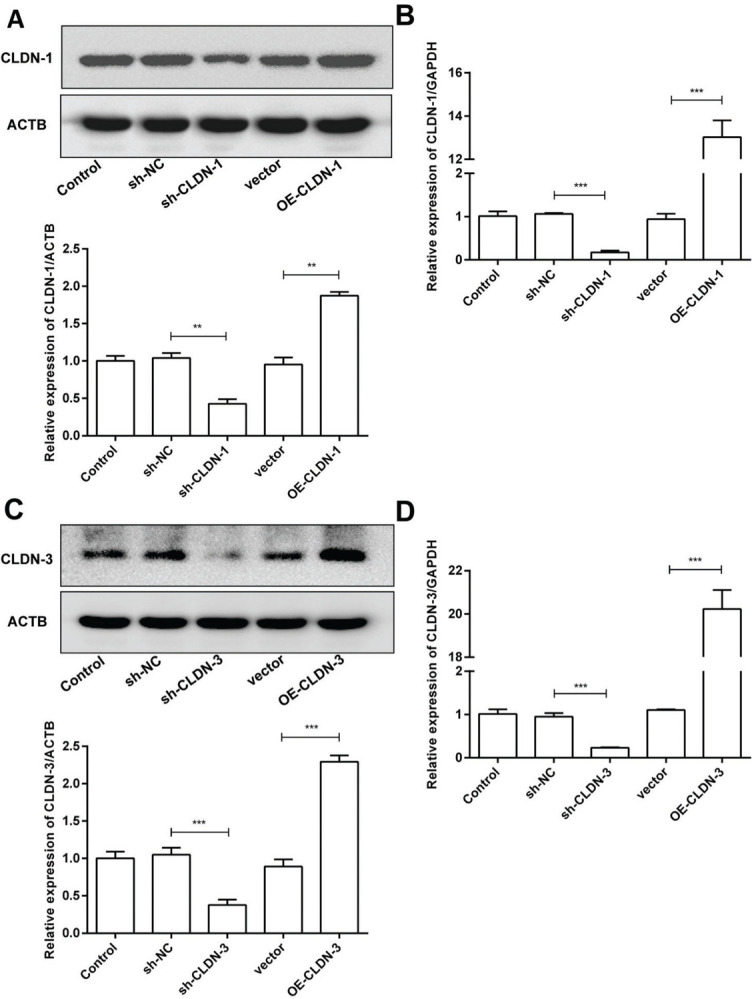
Silencing and over-expression of CLDN-1 and CLDN-3. (A, B) The expression level of CLDN-1 was measured in Oli-neu cells transfected with sh-CLDN-1 and pcDNA3.1-CLDN-1, compared with that of the controls ACTB and GAPDH, respectively. (C, D) The expression level of CLDN-3 was measured in Oli-neu cells transfected with sh-CLDN-3 and pcDNA3.1-CLDN-3, compared with that of the controls ACTB and GAPDH, respectively. **p<0.01 and ***p<0.001 *versus* the respective controls.

**Figure 3:**
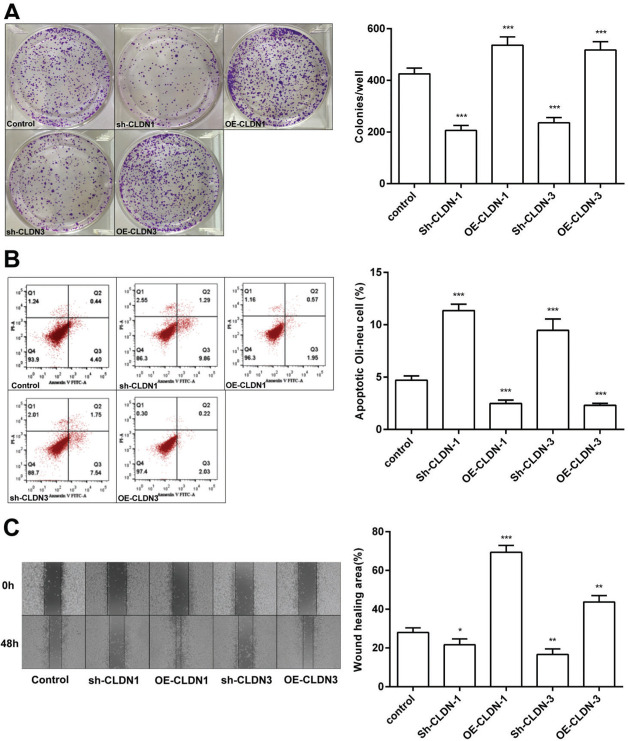
CLDN-1 and CLDN-3 influence the function of Oli-neu cells. (A) The colony-formation ability of Oli-neu cells upon downregulation or overexpression of CLDN-1 or CLDN-3 was determined by the colony formation assay. (B) The detection of apoptosis in Oli-neu cells upon downregulation or overexpression of CLDN-1 or CLDN-3 was performed by Annexin V-FITC staining and flow cytometry. (C) The wound healing area was examined in Oli-neu cells showing the downregulation or overexpression of CLDN-1 or CLDN-3. *p<0.05, **p<0.01, and ***p<0.001 *versus* the respective control.

To examine the role of CLDN-1 and CLDN-3 in oligodendrocytes, we first downregulated and upregulated the expression of CLDN-1 and CLDN-3 in Oli-neu cells (Figure 2). Subsequently, a colony formation assay performed with the transfected Oli-neu cells. The results of this assay revealed that the colony-forming ability of Oli-neu cells is suppressed upon silencing both CLDN-1 and CLDN-3 and enhanced following the overexpression of CLDN-1 and CLDN-3 (Figure 3A). Next, the effects of CLDN-1 and CLDN-3 on the level of apoptosis in Oli-neu cells were further studied by using Annexin V-FITC staining and subsequent flow cytometry analysis. As can be interpreted from Figure 3B, cell apoptosis in Oli-neu cells is significantly stimulated upon CLDN-1 and CLDN-3 downregulation and remarkably inhibited upon the CLDN-1 and CLDN-3 upregulation. Finally, a wound healing assay was performed to detect the effects of alterations in the expression of CLDN-1 and CLDN-3 on the migration ability of Oli-neu cells. Our results revealed that Oli-neu cells with silenced CLDN-1 and CLDN-3 expression demonstrated a lower degree of wound closure than the control cells, and those overexpressing these genes exhibited a higher degree of wound closure (Figure 3C).

### CLDN-1 and CLDN-3 modulate MBP, OLIG2, PLP, and SOX10 expression

**Figure 4:**
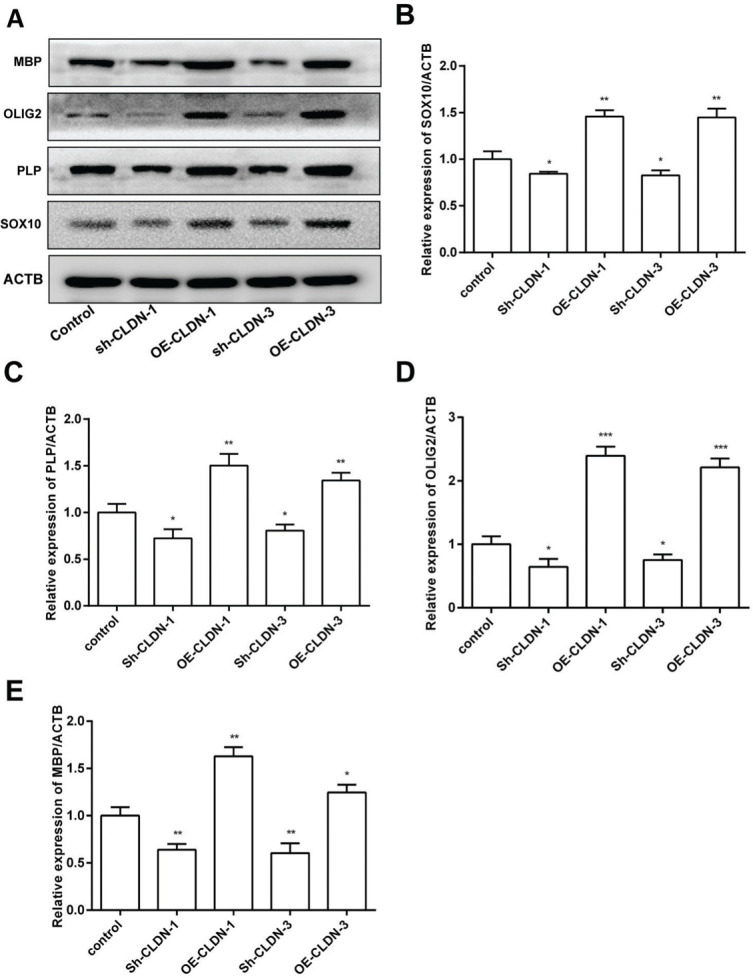
The protein levels of the myelinogenesis-related genes following the downregulation or overexpression of CLDN-1 or CLDN-3 were examined by western blotting. The expression of MBP, OLIG2, PLP, and SOX10 in Oli-neu cells showing the downregulation or overexpression of CLDN-1 and CLDN-3. *p<0.05, **p<0.01, and ***p<0.001 *versus* the respective controls.

To investigate the influence of CLDN-1 and CLDN-3 on myelination, the expression levels of myelination-associated proteins such as MBP, OLIG2, PLP, and SOX10 were examined by western blotting. As shown in Figure 4, silencing CLDN-1 and CLDN-3 notably suppressed the expression levels of MBP, OLIG2, PLP, and SOX10. On the contrary, the overexpression of CLDN-1 and CLDN-3 remarkably enhanced their relative levels. These results indicate that CLDN-1 and CLDN-3 potentially play a noteworthy role in myelinogenesis in Oli-neu cells.

## DISCUSSION

LA is a common phenomenon in patients with ischemic stroke and has been characterized to be closely related to the increased risk of stroke and dementia. It is detected as a non-specific image signal in cerebrovascular diseases and could be examined through MRI with high signals on the lateral ventricle or subcortical area (18,19). The pathogenesis of LA usually involves several factors, including advanced age, history of hypertension, smoking, and other characteristics, and these risk factors are also the main reason for severe symptoms and poor prognosis caused by stroke (20,21). Despite successful thrombectomy for recanalization, the morbidity and mortality rate of patients with this disorder is still remarkably high. To date, the mechanism underlying the pathogenesis of LA has not been completely elucidated. Therefore, it is necessary to investigate the pathogenesis of LA for its early diagnosis and treatment. In this study, we involved 122 patients with Chinese ethnicity exhibiting typical clinical symptoms and brain MRI features for LA. All these patients exhibited a significant cognitive dysfunction, with a lower score of MMSE and MOCA and a higher grade of Fazeka, and presented a strong significant association with lower TC and LDL-C, and higher Hcy levels (Table 1).

CLDN-1 and CLDN-3 are the key components of tight junctions in blood brain barrier, and their abnormal function is one of the main reasons for the occurrence and development of nervous system diseases (22,23). It has been clearly recognized that CLDN-1 transcriptional variants play an important role in LA regulation (24). In particular, the abnormally high expression CLDN-1 and CLDN-3 could modulate tumor proliferation, epithelial-mesenchymal transition, and metastasis capability, et al (25-27). In this study, for the first time, CLDN-1 and CLDN-3 were discovered to exhibit downregulated expression levels in LA patients and remarkably influencing oligodendrocyte growth, migration, and apoptosis. Oligodendrocytes serve as the myelinating cells of the central nervous system during development and throughout adulthood. They are formed due to the activation, proliferation, migration, and differentiation of oligodendrocyte progenitor cells (OPCs). In LA, the complex pathological process results in the dysfunction and apoptosis of oligodendrocytes, leading to demyelination and neurodegeneration (28). These findings indicate that CLDN-1 and CLDN-3 serve as critical factors in regulating oligodendrocyte functions.

Of notable interest was the influence of regulating the CLDN-1 and CLDN-3 expression on the expression of myelinogenesis-related proteins. Our data exhibited that the downregulation of CLDN-1 and CLDN-3 notably represses the expression of MBP, OLIG2, SOX10, and PLP. These four genes serve as the high-risk factors in brain diseases. MBP is the second highest protein in the myelin sheath of central nervous system and is an important part of oligodendrocytes and myelin sheath mainly involved in the actin and tubulin cytoskeleton (29,30). MBP accumulation promotes the production of TNF-related apoptosis-inducing ligand and other pro-inflammatory cytokines, leading to the progressive apoptosis of oligodendrocytes and myelin loss (31). As a neuro-multifunctional regulator, OLIG2 is only expressed in the central nervous system and plays a role in brain-associated diseases (32). OLIG2 expression enhances the differentiation and proliferation of OPCs, improving oxygen/glucose deprivation-induced white matter lesions (33). White matter damage is characterized by demyelination due to the damage of the oligodendrocytes. The continuously expressed SOX10 is particularly important for the terminal differentiation of OPCs (34). Mature oligodendrocytes generate myelin in the central nervous system and thus, ensure the rapid propagation of neuronal activity. PLP is a negative regulator of centrosome maturation and activity (35). The abnormal metabolism of PLP results in oligodendrocyte apoptosis (36). In this study, the expression levels of MBP, OLIG2, SOX10, and PLP were shown to be regulated by CLDN-1 and CLDN-3; this implies that CLDN-1 and CLDN-3 may serve as critical regulatory factors in the central nervous system.

Here, our results show that the expression of CLDN-1, CLDN-3, MBP, OLIG2, and PLP are diminished in LA patients, and SOX10 expression is augmented. Persistent cerebral hypoperfusion induces severe oligodendrocyte death, myelin loss, and activation of inflammation, leading to LA and cognitive impairment (37). Ischemia and hypoxia inhibit the expression of CLDN-1 and CLDN-3, which can induce neuro-inflammation and destroy the blood-brain barrier (14,38). Oligodendrocytes and OPCs are known to be highly sensitive to ischemia and hypoxia (39,40). This vulnerability of oligodendrocytes is a contributing factor to white matter dysfunction. Long-term hypoperfusion inhibits the expression of CLDN-1 and CLDN-3, leading to the destruction of the intercellular barrier and damage to the cells (13,14); this is not conducive to cell proliferation, adhesion, and development (41-43). Oligodendrocytes originate from OPCs, which require the ability to proliferate, migrate, and differentiate into mature myelinating oligodendrocytes (44). It is reported that the numbers of MBP-positive oligodendrocytes and NG2-(Neuron/Glia Antigen 2)-positive OPCs gradually decrease with the aggravation of cerebral ischemia (44). Therefore, the downregulation of CLDN-1 and CLDN-3 expression in LA patients may induce the degeneration and apoptosis of oligodendrocytes and inhibit the development and maturation of OPCs. Severe loss of oligodendrocytes and dysplasia of OPCs results in extensive white matter degeneration or loss. Our study revealed that the knockdown of CLDN-1 or CLDN-3 could inhibit myelinogenesis. The expression levels of myelin proteins such as PLP and MBP are significantly reduced in the white matter, and the number of PLP-positive and MBP-positive cells markedly decrease, which result in the reduction of myelin sheath thickness, and subsequently, LA (45,46). The molecular mechanism underlying the basic promoter activity of PLP and MBP depend on AKT activation (47,48), and CLDN-1 or CLDN-3 knockdown can significantly inhibit AKT activity (41,49). Therefore, the reduction of the expression levels of CLDN-1 and CLDN-3 in LA patients may attenuate MBP and PLP synthesis by downregulating AKT signaling. Besides, the increased levels of phosphorylated-AKT promotes Wnt/β-catenin/Tcf4 signal transduction; this inhibits OLIG2, SOX10, PLP, and MBP expression in OPCs (46). The differentiation or maturation of OPCs is regulated by the transcription factors OLIG1, OLIG2, NKX2.2, and SOX10 (50). The decreased expression of OLIG2 and SOX10 impedes OPC differentiation and maturation. However, the level of SOX10 is found to be increased in LA patients; this may be related to the heterogeneity of lesion development in the white matter (51). In short, our results suggest that the downregulation of CLDN-1 and CLDN-3 inhibits oligodendrocyte viability and OPC compensation, leading to axonal hypomyelination, white matter lesions, and cognitive dysfunction.

In conclusion, this study demonstrated that CLDN-1 and CLDN-3 are critical factors in patients with LA. Silencing CLDN-1 and CLDN-3 inhibits cellular growth and migration and promotes apoptosis via regulating the expression of myelinogenesis-related proteins such as MBP, OLIG2, PLP, and SOX10. These results may provide novel insights into the regulatory role of CLDN-1 and CLDN-3 in the pathogenesis of LA.

## AUTHOR CONTRIBUTIONS

Chen Y and Zheng Z made substantial contributions to the conceptualization and design of the study. Chen Y, Zheng Z, Mei A, Huang H and Lin F made substantial contributions to the acquisition, analysis and interpretation of data for the study. Chen Y participated in the manuscript drafting, while Zheng Z and Mei A were involved in revising the manuscript critically with respect to important intellectual interpretations. Each author approved the final version of the manuscript to be published and agreed to be accountable for all aspects of the study, ensuring that questions related to the accuracy or integrity of any part of it are appropriately investigated and resolved.

## Figures and Tables

**Table 1:** Patients’ clinical characteristics.

Factors	NC (n=122)	LA (n=122)	*p*-value
Age (years)	71 (66.75-76)	72 (67-77)	0.155a
Male (%)	64 (52.46%)	75 (61.48%)	0.374b
Female (%)	58 (47.54%)	47 (38.52%)	
CRP (mg/L)	3.5 (1.67-5.81)	3.05 (1.4-6.7)	0.73a
TC (mmol/L)	4.76 (4.16-5.35)	4.28 (3.55-4.85)	<0.001a
LDL-C (mmol/L)	2.98 (2.39-3.63)	2.67 (2.28-3.14)	0.005a
HDL-C (mmol/L)	1.04 (0.87-1.24)	1.02 (0.87-1.2)	0.964a
GLU (mmol/L)	5.38 (4.61-6.48)	5.55 (4.90-6.58)	0.189a
TG (mmol/L)	1.62 (1.21-1.99)	1.53 (1.2-1.78)	0.045a
UA (mmol/L)	331 (273-414)	377.5 (320-412.25)	0.029a
HbA1c (mmol/L)	6.5 (5.9-7.23)	6.2 (5.8-6.86)	0.051a
Hcy (mmol/L)	9.82 (8.79-11.02)	11.75 (9.67-13.72)	<0.001a
MMSE Grade	28 (26-29)	21 (18-25)	<0.001a
MoCA Grade	25 (23-27)	20 (16-23.25)	<0.001a
Fazeka Grade	0 (0-0)	2 (1-3)	<0.001a

Abbreviations: NC, Normal control; LA, Leukoaraiosis; CRP, C-reactive protein; TC, total cholesterol; LDL-C, low-density lipoprotein cholesterol; HDL-C, high-density lipoprotein cholesterol; GLU, Fasting blood glucose; TG, triglyceride; UA, Uric acid; HbA1c, Glycosylated hemoglobin; Hcy, Total plasma homocysteine; MMSE, the Mini-Mental State Examination; MoCA, Montreal cognitive assessment scale. Data are presented as the number and percentage (%) and median with interquartile interval [Q1-Q3]. a, Mann-Whitney U test; b, Chi-square test. *p*<0.05 was considered to suggest a significant difference.

**Table 2:** Primers for RT-PCR.

Gene	Forward primer	Reverse primer
CLDN-1	TTGGGCTTCATTCTCGCCTT	GTCGCCGGCATAGGAGTAAA
CLDN-3	ACGCGAGAAGAAGTACACGG	GTAGTCCTTGCGGTCGTAGC
MBP	GGCAAGGTACCCTGGCTAAA	TGTACATGTTGCACAGCCCA
PLP	TGCTGTGCAAGATGTCTGGT	AACAGTGCCACCCCAAAGAA
OLIG2	TCGCATCCAGATTTTCGGGT	GCAGAAAAAGGTCATCGGGC
SOX10	ACAAGAAAGACCACCCGGAC	AAGTGGGCGCTCTTGTAGTG
GAPDH	CATGTTGCAACCGGGAAGGA	CAGGAGCGCAGGGTTAGTC
